# A Comparison of Esthetic Preferences on Female Skeletal Class II Alterations among Laypeople of Different Facial Profiles

**DOI:** 10.1055/s-0044-1788654

**Published:** 2024-07-29

**Authors:** Wiwan Tipyanggul, Chidsanu Changsiripun, Niramol Chamnannidiadha

**Affiliations:** 1 Department of Orthodontics, Faculty of Dentistry, Chulalongkorn University, Henri-Dunant Road, Wangmai, Pathumwan, Bangkok, Thailand

**Keywords:** esthetics, laypeople, photograph, silhouette, skeletal Class II

## Abstract

**Objectives** This study aimed to investigate the influences of assessors' different personal profiles on the esthetic perception of Class II facial profile corrections and the agreement between profile and silhouette images.

**Materials and Methods** A profile photo of a female with skeletal Class II was digitally altered into three profile and three silhouette images (most pronounced Class II division 1 characteristic, more retruded upper lip position, and more protruded mandibular position). Ninety-six laypeople from three facial profile groups (straight, convex, and concave profiles) chose these images for facial attractiveness. Data were analyzed using an SPSS program. Cohen's kappa coefficient and intraclass correlation coefficients were applied to determine intraparticipant and intra-examiner reliabilities. Chi-square tests were used to test between-group preferences and the relationship of profile preference with other factors. Cohen's kappa coefficient was used to test the agreement in selecting profile and silhouette images ( *p* = 0.05).

**Results** All groups favored profiles with a protruded mandibular position (11-degree facial contour angle [FCA] and 91-degree nasolabial angle [NLA]). Despite facial profile differences, preference remained consistent ( *p* = 0.649). The convex group showed a stronger inclination toward an untreated-simulating profile (17-degree FCA and 91-degree NLA). Preferences were consistent regardless of sex ( *p* = 0.198) and education ( *p* = 0.105). The percentage of agreement between profile and silhouette images in the total sample was 67.71% (kappa = 0.386). All groups of participants chose the more retruded upper lip position (17-degree FCA and 107-degree NLA) profile in silhouette more than in photograph.

**Conclusion** All groups preferred a mandibular advancement-simulating profile. Using the photographs or silhouettes to assess the esthetic preference resulted in a similar trend. However, the flatter profile was more preferred in silhouette than in photograph.

## Introduction

Orthodontic practices have undergone significant transformations in clinical evaluation, diagnosis, and treatment planning over time. Presently, a key focus lies in enhancing facial esthetics as a primary treatment goal. 1 2 3 Patients pursue orthodontic treatment for various reasons, with psychosocial concerns being a significant motivator. Particularly, young adults often seek treatment to enhance their appearance or improve facial or dental imperfections because enhancement of facial and dental appearance can have a major impact on improving the quality of life. 1 4 5

When addressing Class II patients, treatment options may involve growth modification, orthognathic surgery, or utilizing dental compensation techniques to conceal skeletal discrepancies. While all approaches lead to enhancements in the sagittal interlabial step and as well as the harmonious beauty of the face, they target different facial features and yield distinct outcomes, particularly in terms of facial esthetics. 1 6 The camouflage treatment in skeletal Class II typically involves retracting the maxillary incisors, resulting in an increased nasolabial angle (NLA). 7 8 Conversely, surgical interventions aim to improve skeletal problem through methods of mandibular advancement, maxillary setback, or both. These surgical corrections also affect the facial profile by decreasing the facial contour angle (FCA). 9 10 Studies indicate that both of these changes are considered attractive. 7 8 9 10 Moreover, esthetic medicine plays an important role in today's treatments. Soft tissue filler injections in the chin area enhance the likelihood of patient satisfaction, 11 12 13 which is crucial in facilitating camouflage treatments.

The studies that investigated the influence of assessors' personal profiles on esthetic perception are small in number and inconsistent. Some of the studies found similarities in facial esthetic perception with a slight difference among each facial profile group. 9 14 15 But there was a study that found differences in esthetic perception of adults with different facial profiles. 16 Same researchers also found that adolescents (13–18 years) and adults with straight profiles were more satisfied with their profiles than those with convex and concave profiles. However, there was no study focusing on the esthetic perception of the Class II corrections.

The tools commonly used for assessing esthetic preferences in the profile view are the patient's photograph or the silhouette. 17 The patient's photograph was suggested to be used because the patient's facial appearance can be illustrated more than the silhouette, 18 19 and using the silhouette could cause the clinician to select the profile differently from the esthetic norm. 19 However, some researchers have used the silhouette to remove the influence of other factors such as eye, skin complexion, and hair color that might bias the perception of facial attractiveness. 20 21

This study aims to investigate (1) the influences of assessors' personal profiles on the esthetic perception of Class II facial profile corrections and (2) the agreement of esthetic perception in selecting between the profile and silhouette images. The results of the study can be used as part of the data to establish treatment planning and decision-making processes, and can also be utilized in future studies.

## Materials and Methods

### Sample

**Fig. 1:**
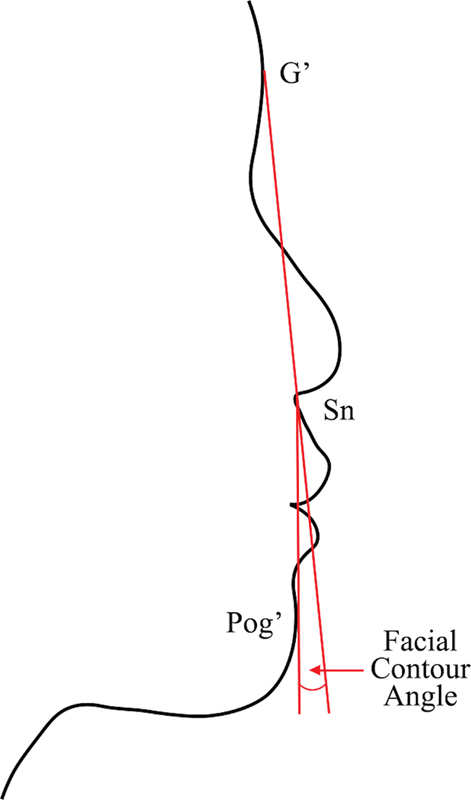
Facial contour angle: an angle defined by soft tissue glabella (G′), subnasale (Sn), and soft tissue pogonion (Pog′).

The estimated sample size was calculated by using the n4Studies application (version 1.4.1), 22 using the formula for testing the infinite population proportion, *n* = ( *z* ^2^_1 − α/2_ *p* [1 − *p* ])/ *d* ^2^ , with *p* = 0.571, *d* = 0.1, and α = 0.05 to detect the posttreatment preference of the laypeople. 8 The results indicated that a total of 95 participants were needed. The participants consisted of 96 Thai laypeople randomly picked from Chulalongkorn university dental clinic, shopping malls, and educational institutions in Bangkok, Thailand, categorized into three groups based on their FCAs 23 : straight (FCA 5–13 degrees), convex (FCA >13 degrees), and concave (FCA <5 degrees). Each group consisted of 32 participants with a 1:1 male-to-female ratio. Exclusion criteria were participants who are dental professionals (consisting of dentists, dental students, dental assistants, and dental hygienists), younger than 16 years, older than 40 years, with a history of facial trauma, conditions with syndromes, or serious medical conditions. The measurement of participants' FCA was done by taking a photo of each participant in the right nonsmiling profile view with the teeth in centric occlusion and lips relaxation. The participants were positioned 5 feet from the camera with their heads in a natural posture. Measurement of the FCA was done by a single researcher (W.T.), using Adobe Photoshop 2020 (Adobe Systems Inc., San Jose, California, United States). The FCA is defined by the points: soft tissue glabella (G′), subnasale (Sn), and soft tissue pogonion (Pog′) as illustrated in Fig. 1 . Twenty percent of the participants' FCA (19 participants) were measured again at least 4 weeks after the initial assessment to determine the reliability of the examiner.

### Photo Album

A photograph capturing the right nonsmiling profile view was taken of a female subject presenting the following characteristics: an untreated skeletal Class II relationship with orthognathic maxilla, retrognathic mandible, straight forehead, straight nose dorsum, normal vertical proportion, and a normal mandibular plane angle. 23 The subject was positioned at a distance of 5 feet from the camera, maintaining a natural head posture. Permission was granted by the subject for the photograph to be taken, the image to be adapted, and for online publication.

The profile picture was initially modified with Adobe Photoshop 2020 (Adobe Systems Inc.) to accentuate the mandibular retrusion. This was achieved by increasing the FCA by 2 standard deviations (SD), resulting in a 17-degree FCA, using the Thai FCA norm of 9 ± 4 degrees as indicated by Sorathesn. 23 Additionally, the NLA was adjusted according to the Thai NLA norm of 91 ± 8 degrees, 23 resulting in a 91-degree NLA. Hence, the “base image” used for further modifications depicted a profile with a 17-degree FCA and a 91-degree NLA, representing the largest sagittal interlabial step.

Two additional alteration images were created, using the “base image” in Photoshop, focusing on changes in the anteroposterior plane while maintaining vertical proportion. In the first image, the soft tissue Pog′ point was advanced to reduce the FCA by 1.5 SD, resulting in an 11-degree FCA. 23 24 25 Additionally, the mentolabial sulcus was adjusted in a 1:1 ratio following the movement of the soft tissue Pog′. In the second image, the labrale superius point (Ls point) was retruded to increase the NLA by 2.0 SD, resulting in a 107-degree NLA. 7 23 26

**Fig. 2:**
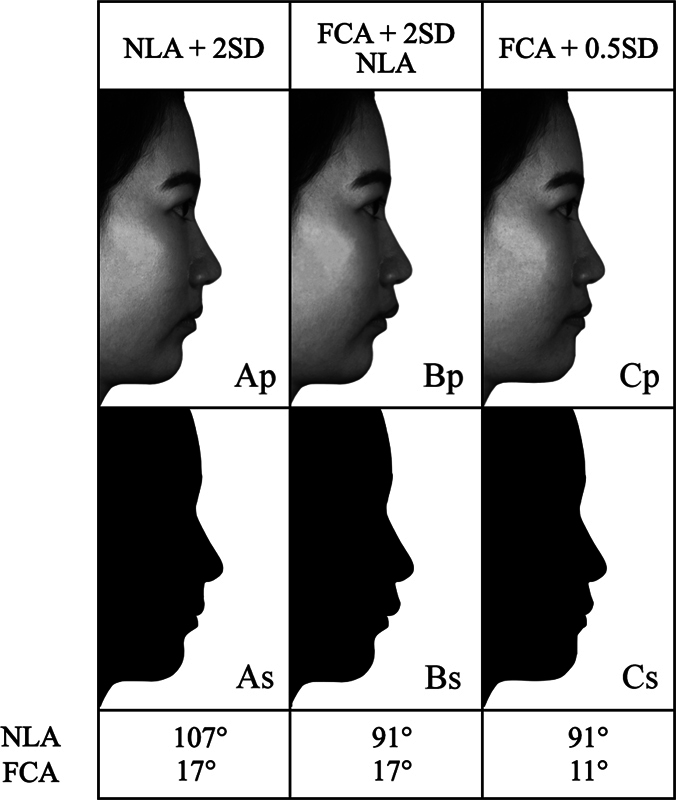
Altered profile and silhouette images; most pronounced Class II division 1 characteristic (Bp and Bs), more retruded upper lip position (Ap and As), and more protruded mandibular position (Cp and Cs). FCA, facial contour angle; NLA, nasolabial angle; SD, standard deviation. (Adapted with permission from Tipyanggul et al. 31 )

In summary, three altered profiles ( Fig. 2 ; images Ap, Bp, and Cp) were created. One depicted the most pronounced Class II division 1 characteristic, with a 17-degree FCA and 91-degree NLA which was an untreated-simulating profile ( Fig. 2 ; image Bp). Another simulated a more retruded upper lip position which was a camouflage-simulating profile ( Fig. 2 ; image Ap). The third simulated a more protruded mandibular position which was a mandibular advancement-simulating profile ( Fig. 2 ; image Cp). Subsequently, all three altered profile images were converted to black and white ( Fig. 2 ; images Ap, Bp, and Cp) and modified to produce the silhouette images ( Fig. 2 ; images As, Bs, and Cs).

All three altered profile images were simultaneously presented to the participants using the photos application on iPad Pro 10.5-inch (Apple Inc., Cupertino, California, United States). Three profile images were placed alongside each other. The base image with the most pronounced Class II division 1 characteristic ( Fig. 2 ; image Bp) was placed in the center, the image with an increased NLA ( Fig. 2 ; image Ap) was on the left, and the image with a decreased FCA ( Fig. 2 ; image Cp) was on the right.

The first page of the photo album showed images as depicted in Fig. 2 ; images Ap, Bp, and Cp. Pages 2 and 3 featured the same image set but with positions randomly arranged to create a washout effect. Page 4 displayed the silhouette images as in Fig. 2 ; images As, Bs, and Cs, also in randomly arranged positions. These pages, from 1 to 4, were utilized to fulfill Part 2 of the questionnaire.

Twelve participants (four per group) were asked to reassess the altered profile images at least 4 weeks after the initial assessment to determine the reliability of the test.

### Questionnaire

In Part 1 of the questionnaire, participants provided demographic details including age, sex, ethnicity, and level of education. Part 2 required participants to view each page of a photo album. The participants were also asked, in the form of a closed-ended question, to select the profile image they found most attractive in terms of facial appearance. Participants were given 60 seconds to complete the assessment for each page and were asked not to go back to the page they had already assessed. The questionnaire utilized in this study is available in Supplementary Materials S1 (available in the online version only).

### Statistical Analysis

The statistical analysis was conducted using SPSS version 22.0 for Mac (IBM, Chicago, Illinois, United States). Intraparticipant and intra-examiner reliabilities were evaluated by calculating Cohen's kappa coefficient and intraclass correlation coefficients, respectively. The profile preference among laypeople of different facial profiles (between-group preference) was tested using chi-square tests. The relationship of the profile preference with other factors (sex and level of education) was analyzed using chi-square tests. The agreement in selecting the profile and silhouette images was assessed with Cohen's kappa coefficient. A significance level of 0.05 was set for all tests.

## Results

### Demographic Data

Ninety-six participants were stratified into three groups ( *n* = 32): straight, convex, and concave. Each group had an equal male-to-female ratio of 1:1. Table 1 presents the baseline data for the three groups, including varying levels of education within each group.

**Table 1:** Baseline data of the three profile groups

	Group		
	Straight	Convex	Concave
Age median (Q1, Q3)	21.89 (18.23, 25.49)	22.31 (18.87, 25.91)	22.39 (20.29, 26.11)
Level of education ( *n* )			
Nongraduate	14 (43.8%)	13 (40.6%)	8 (25%)
University graduate	18 (56.2%)	19 (59.4%)	24 (75%)

### Reliability Coefficients of Intraparticipants and Intra-examiner

The intrarater reliability of the participants in the profile and silhouette images was substantial (0.692 and 0.667, respectively). The reliability of the examiner was excellent (0.999).

### Profile Preference among Laypeople of Different Facial Profiles

The chi-square test did not show a significant difference in the profile preference among laypeople of different facial profiles ( *p* = 0.649) ( Table 2 ). Image Ap was chosen by 25% of the straight group, 12.5% of the convex group, and 21.9% of the concave group. Image Bp was chosen by 9.4% of the straight group, 18.8% of the convex group, and 12.5% of the concave group. Image Cp was chosen by 65.6% of the straight group, 68.8% of the convex group, and 65.6% of the concave group.

**Table 2:** Profile preference among laypeople of different facial profiles (between-group difference)

Preference	Group		
*N* (%)	Straight	Convex	Concave
Ap	8 (25%)	4 (12.5%)	7 (21.9%)
Bp	3 (9.4%)	6 (18.8%)	4 (12.5%)
Cp	21 (65.6%)	22 (68.8%)	21 (65.6%)
Pearson's chi-square value = 2.477 *p* -Value = 0.649			

### Profile Preference among Laypeople within the Same Facial Profile

**Fig. 3:**
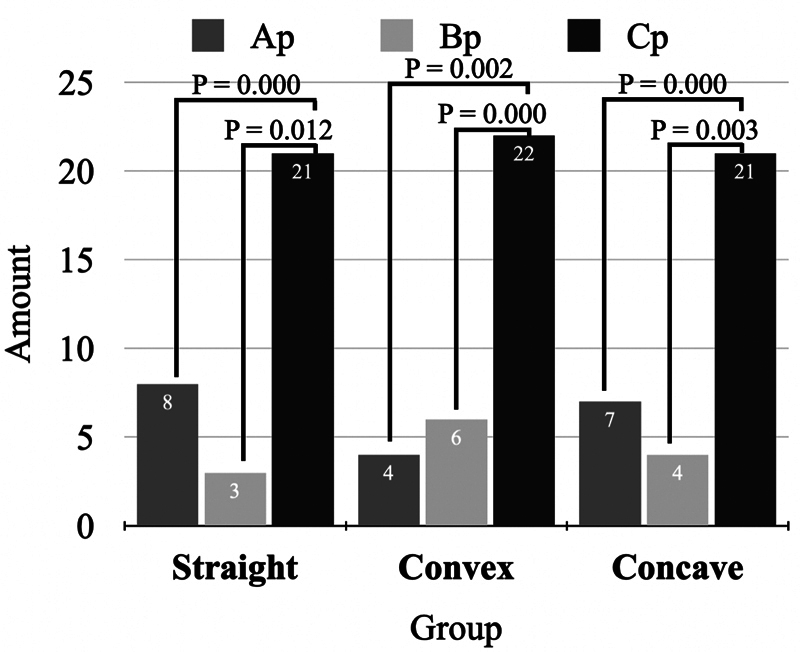
Profile preference among laypeople within the same facial profile (within-group difference). (Ap—17-degree FCA and 107-degree NLA, Bp—17-degree FCA and 91-degree NLA, and Cp—11-degree FCA and 91-degree NLA). FCA, facial contour angle; NLA, nasolabial angle.

The most chosen profile for all 96 participants was a more protruded mandibular position profile (image Cp) (straight group: 65.6%, convex group: 68.8%, and concave group: 65.6%), followed by a more retruded upper lip position profile (image Ap) in the straight (25%) and concave (21.9%) groups. While the convex group preferred the most pronounced Class II division 1 profile (image Bp) (18.8%) more than image Ap. The significant differences were found between images Cp–Ap and images Cp–Bp ( Fig. 3 ).

### The Relationship of the Profile Preference with Other Factors

According to the chi-square test, the profile preference did not differ by sex ( *p* = 0.198) and level of education ( *p* = 0.105).

### The Agreement in Selecting the Profile and Silhouette Images

The profile preferences for profile and silhouette images are shown in Table 3 . The percentage of agreement between the methods in the total sample was 67.71%, which could be considered as a fair agreement (kappa = 0.386). A more protruded mandibular position profile (image C) was the most chosen in both profile and silhouette images.

**Table 3:** The agreement in selecting the profile and silhouette images

Preference	Images	
*N* (%)	Profile	Silhouette
A	19 (19.8%)	27 (28.1%)
B	13 (13.5%)	11 (11.5%)
C		58 (60.4%)
	64 (66.7%)	
Kappa = 0.386 Percentage of agreement = 67.71%		

## Discussion

The present study investigated the influence of the assessors' facial profile on the esthetic perception of Class II facial profile corrections. The results demonstrated that the esthetic preference among laypeople with different facial profiles was similar. There was no significant difference in the profile preference among laypeople of different facial profiles. Volpato et al 15 reported that the pleasantness scores assigned by the patients of three facial profile types were not different, although the patients with straight profile assigned slightly greater scores than patients with concave or convex profiles. Suphatheerawatr and Chamnannidiadha 9 also found that participants with different facial profiles had similar facial profile preferences.

Image Cp with normal NLA (11-degree FCA and 91-degree NLA) was the most chosen profile for all three groups of laypeople, followed by image Ap with obtuse NLA (17-degree FCA and 107-degree NLA) in the straight and concave groups. The least chosen profile for the straight and concave groups was image Bp with the most pronounced Class II division 1 profile (17-degree FCA and 91-degree NLA). While the convex group preferred image Bp more than image Ap. These results indicated that the convex group of laypeople preferred the convex profile more than other groups. Jarungidanan and Sorathesn 14 reported that the subject with a convex profile accepted convex profiles equally or more than any other profile subjects. Suphatheerawatr and Chamnannidiadha 9 also reported that an extremely convex profile was preferred by the convex group of the assessors more than the concave group of the assessors.

Moreover, the images Ap and Cp were the profile images that were intended to simulate the Class II treatment which was a camouflage-simulating profile and mandibular advancement-simulating profile, respectively. These two profile images were chosen more than image Bp which was the most pronounced Class II division 1 characteristic (untreated-simulating profile). These results indicated that treating Class II patients with dental compensation or orthognathic surgery has different effects on attractiveness according to laypeople. Camouflage treatment for skeletal Class II typically involves retracting the maxillary incisors, which increase the NLA. 7 8 On the other hand, surgical interventions aim to correct skeletal problems through methods such as mandibular advancement, maxillary setback, or both. These surgical corrections also influence the facial profile by reducing the FCA. 9 10 Yüksel et al 27 also reported that the untreated profile was found to be least preferred and the mandibular advancement and camouflage treatment were considered more attractive than the untreated profile with the mandibular advancement profiles more attractive than the camouflage treatment profiles.

This study found the fair agreement in selecting the profile and silhouette images. Participants tended to choose the profile and silhouette images in similar trend. The most chosen profile was image Cs, followed by image As, and the least chosen was image Bs. However, the number of the participants that chose image Cs was less in silhouette images with the increasing number of the participants that chose image As. These results showed that the profile flatter than the esthetic norm was more preferred in silhouette than in photograph. Hockley et al 19 also found that using the photographs in the profile esthetic assessment, the participants preferred the photographs that closer the esthetic norm more than using silhouettes. While using the silhouettes, the participants tended to select the flatter profile than the esthetic norm.

This study aimed to isolate the factors influencing esthetic perception. Thus, all three profiles were adjusted to achieve normal vertical proportions 21 and a straight nose dorsum. Prior research has shown that a straight nose dorsum is perceived as more esthetically pleasing, as opposed to differing nose shapes in Class II profiles. 28 Therefore, variations in vertical proportions and nose shapes among Class II patients could yield different esthetic preferences compared with those found in this study. Treatment planning in orthodontics involves considering various factors such as incisor display, gingival display, tooth proportion, gingival shape and contour, and tooth shade. The findings of this study should be regarded as one component among many others in aiding the treatment planning process.

The purpose of altering the profile images in this study was to establish different degrees of NLA and FCA, corresponding to mandibular advancement or camouflage treatment. These adjustments also affected soft tissue in other facial areas, particularly the sagittal interlabial step. Additional images altered from the “base image” displayed varying degrees of sagittal interlabial steps, with less prominence compared with the base image. This was achieved through retrusion of Ls point (image Ap) and the advancement of Pg′ point which led to the advancement of the lower lip (image Cp). Interestingly, the least favored profile image among the straight and concave groups of laypeople was the “base image” Bp, which had the largest sagittal interlabial step. These findings align with Yüksel et al's study, 27 which concluded that reducing the sagittal interlabial step through changes in NLA or FCA increased assessor satisfaction compared with profiles with larger interlabial steps. Moreover, prior research has shown that postoperative changes in the Pg′ point and mentolabial sulcus correlate with changes in underlying hard tissue at a 1:1 ratio. 24 29 30 Thus, in altering image Cp, adjustments to the mentolabial sulcus were made in accordance with these previous findings.

This study was primarily concerned with the personal profiles of participants, with a specific focus on their FCA. Consequently, data collection was centered around assessing this parameter. Acknowledging the influence of age on preferences as the results found in the previous study, 31 participants recruited for this study ranged in age from 16 to 40 years. Varatharaju et al 32 observed that self-recognition of facial profiles tends to improve with age, with individuals older than 15 years showing significantly better recognition compared with younger subjects. Additionally, young adults between the ages of 20 and 39 years often seek orthodontic treatment for esthetic improvement or correction of perceived defects. 1 However, other participant factors were not controlled, making it challenging to regulate sample size across these variables. Our findings revealed no statistically significant differences in profile preference related to sex and level of education, aligning with the results reported by Pithon et al, 17 who found no significant differences based on race, sex, or educational background. To explore these factors further, we recommend future studies collect larger, more evenly distributed samples across these variables. Additionally, this study focused on participants of the same ethnicity to control for this contributing factor, but future research could include participants from diverse ethnic backgrounds for a broader understanding of profile preference trends.

Based on the factors we attempted to control, there are limitations in this study concerning variables such as ethnicity, participant age, vertical image proportion, and nasal shape. As a result, the study's data are applicable clinically as only a part of the decision-making process, rather than as the sole determining factor. Thus, we advocate for future research endeavors to further investigate the controlled factors in this study to enhance clinical knowledge and applicability.

## Conclusion

A mandibular advancement-simulating profile was the most preferred profile for all three groups of participants. There was no significant difference in the profile preference among laypeople of different facial profiles, but the convex group tended to prefer an untreated-simulating profile more than the straight and concave group. The profile preference did not differ by sex and level of education. Using the photographs or silhouettes to assess the esthetic preference resulted in a similar trend with the profile flatter than the esthetic norm was more preferred in silhouette than in the photograph.

Orthodontists and patients can use these findings as part of the data to establish their treatment planning and decision-making processes. However, successful treatment planning in each case requires consideration of numerous factors, such as correct diagnosis, appropriate timing of treatment, and the patient's treatment goals.
