# Supplementary material for: A Comparison of Esthetic Preferences on Female Skeletal Class II Alterations among Laypeople of Different Facial Profiles
Source: Eur J Dent. 2024 Jul 29;19(2):366–73. doi: 10.1055/s-0044-1788654 (PMC12020620; doi:10.1055/s-0044-1788654)
Supplement: Supplementary file 1 — Supplementary Material [file 10-1055-s-0044-1788654-s2453552.pdf]

Supplementary Material S1 Research questionnaire

Instructions

1. This questionnaire aims to investigate the esthetic perceptions from the side profile. It comprises the following details:
   Part 1: General demographic information
   Part 2: Questionnaire on esthetic perceptions
2. Please respond truthfully to the questionnaire, as only accurate and comprehensive responses can maximize the utility of this research.
3. All data collected through the questionnaire will be treated with confidentiality and will be aggregated solely to generate comprehensive reports.
   This questionnaire is part of a research study to investigate the influences of evaluators' personal profiles on the esthetic perception of Class II facial profile corrections. All data collected through the questionnaire will be treated with confidentiality. We ask that all evaluators answer the test truthfully.

Part 1: General demographic information
Instructions: Please mark ✔ in □ or fill in the blanks

1. Date/month/year of birth ……………………………………
   ………………...
2. Sex
   □Male □Female
3. Ethnicity
   □Thai □Others
4. Current address
   □ Bangkok and Metropolitan area (Nakhon Pathom, Nonthaburi, Pathum Thani, Samut Prakan, and Samut Sakhon)
   □ Others ………………………………………………………………………………
   …………
5. Education
   □ Nonuniversity graduate □ University graduate

Part 2: Questionnaire on esthetic perceptions
Instructions:

1. There are four sets of assessments included in this study.
2. Please examine all three profile images in each set.
3. Then, ***select only one profile image*** that you found most attractive in terms of facial appearance *in each set*.
4. Refrain from revisiting or reviewing previous images.
   Please limit your evaluation of each set to no more than 60 seconds.

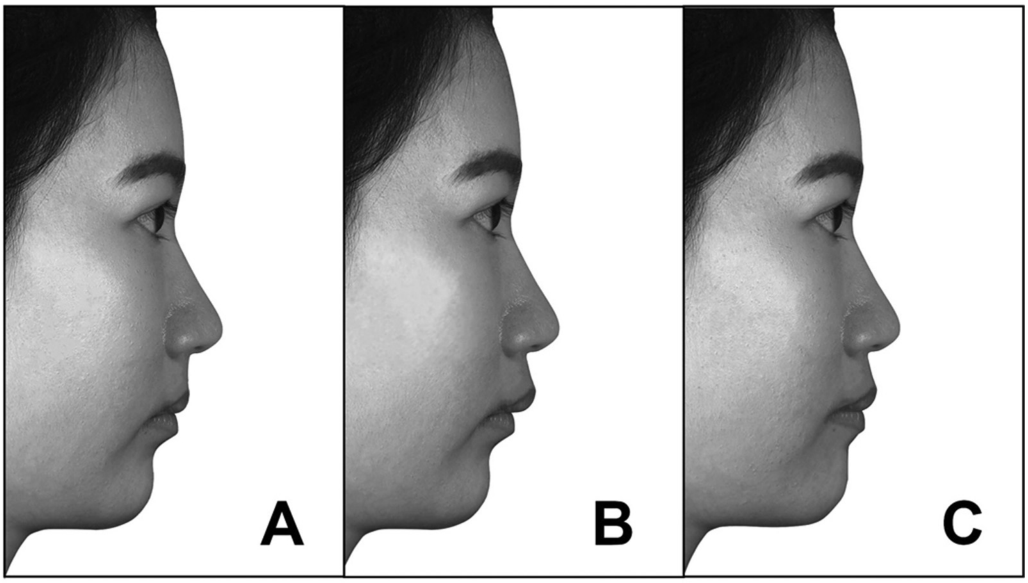

Set 1 □ □ □

 © 2024. The Author(s).

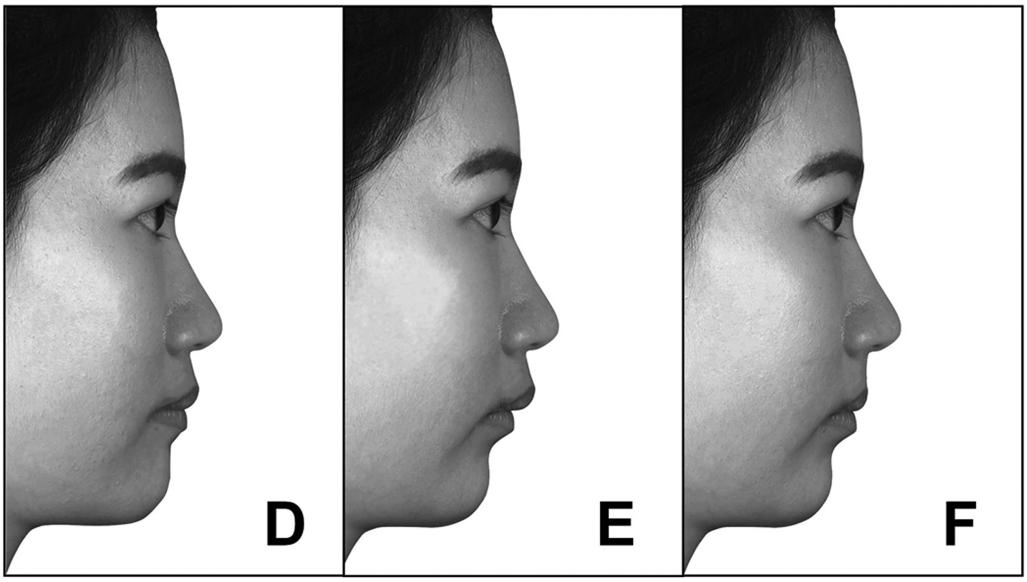

Set 2 ☐ ☐ ☐

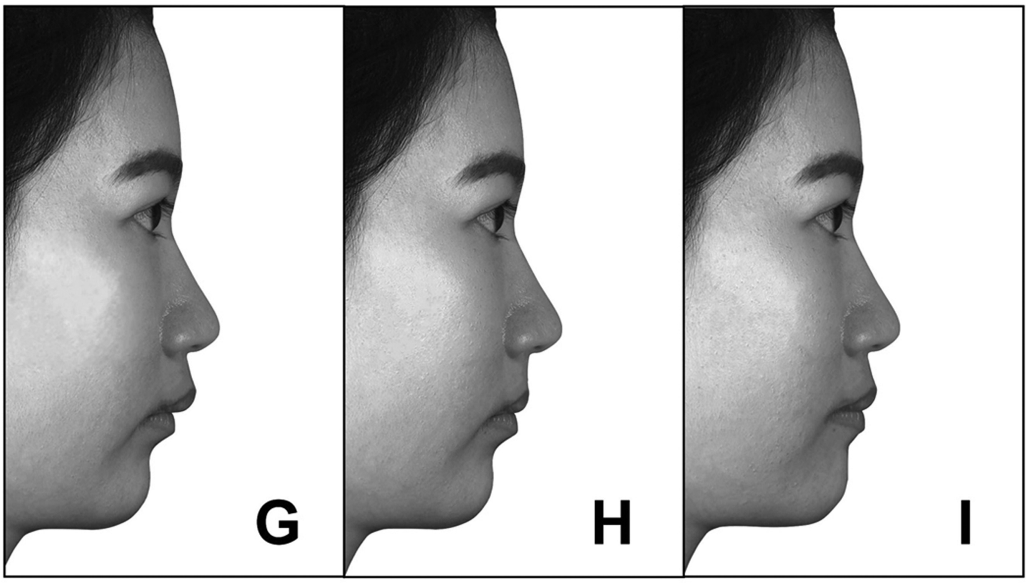

Set 3 ☐ ☐ ☐

 © 2024. The Author(s).

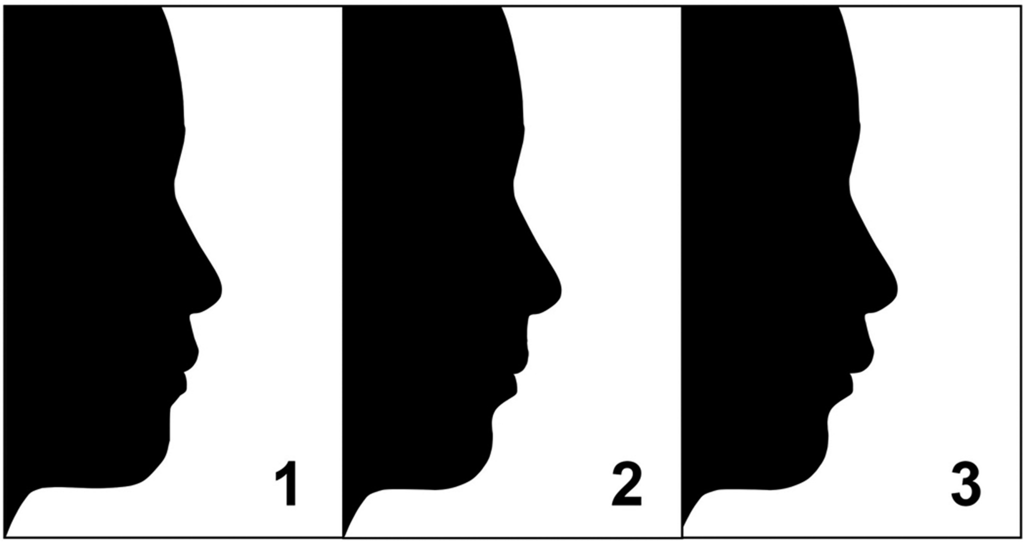

Set 4 ☐ ☐ ☐
